# OA06.02. Impact of Tai Chi exercise on multiple fracture-related risk factors in post-menopausal osteopenic women: a pilot pragmatic, randomized trial

**DOI:** 10.1186/1472-6882-12-S1-O22

**Published:** 2012-06-12

**Authors:** P Wayne, D Kiel, J Buring, P Bonato, G Yeh, C Cohen, C Mancinelli, R Davis

**Affiliations:** 1 Harvard Medical School, Brigham and Women's Hospital, Boston, USA; 2 Institute for Aging Research, Hebrew SeniorLife, Harvard Medical School, Boston, USA; 3 Spaulding Rehabilitation Hospital, Harvard Medical School, Boston, USA; 4 Beth Israel Deaconness Medical Center, Harvard Medical School, Boston, USA; 5 Harvard Vanguard Medical Associates, Internal Medicine, Boston, USA

## Purpose

Tai Chi (TC) is a mind-body exercise that shows potential as an effective and safe intervention for preventing fall-related fractures in the elderly. Few randomized trials have simultaneously evaluated TC’s potential to reduce bone loss and improve fall-predictive balance parameters in osteopenic women.

## Methods

In a pragmatic randomized trial, 86 post-menopausal osteopenic women, aged 45-70, were recruited from community clinics. Women were assigned to either nine months of TC training plus usual care (UC) vs. UC alone. Primary outcomes were changes between baseline and nine months of bone mineral density (BMD) of the proximal femur and lumbar spine (dual-energy X-ray absorptiometry) and serum markers of bone resorption and formation. Secondary outcomes included quality of life. In a subsample (n=16), quiet standing fall-predictive sway parameters and clinical balance tests were also assessed. Both intent-to-treat and per-protocol analyses were employed.

## Results

For BMD, no intent-to-treat analyses were statistically significant; however, per protocol analyses (i.e., only including TC participants who completed ≥ 75% training requirements) of femoral neck BMD changes were significantly different between TC and UC (+0.04 vs. -0.98%; p=0.05). Changes in bone formation markers and physical domains of quality of life were also more favorable in per protocol TC vs. UC (p=0.05). Changes in sway parameters were significantly improved by TC vs. UC (average sway velocity, p=0.027; anterior-posterior sway range, p=0.014). Clinical measures of balance and function showed statistically non-significant trends in favor of TC.

## Conclusion

TC training offered through existing community-based programs is a safe, feasible, and promising intervention for reducing multiple fracture risks. Our results affirm the value of a more definitive, longer-term trial of TC for osteopenic women, adequately powered to detect clinically relevant effects of TC on attenuation of BMD loss and reduction of fall risk in this population.

